# Quantifying the Impact of Phenoconversion on Medications With Actionable Pharmacogenomic Guideline Recommendations in an Acute Aged Persons Mental Health Setting

**DOI:** 10.3389/fpsyt.2021.724170

**Published:** 2021-08-19

**Authors:** Sam Mostafa, Thomas M. Polasek, Leslie J. Sheffield, David Huppert, Carl M. J. Kirkpatrick

**Affiliations:** ^1^Centre for Medicine Use and Safety, Monash University, Parkville, VIC, Australia; ^2^MyDNA Life, Australia Limited, South Yarra, VIC, Australia; ^3^Certara, Princeton, NJ, United States; ^4^Department of Clinical Pharmacology, Royal Adelaide Hospital, Adelaide, SA, Australia; ^5^Department of Genetic Medicine, Melbourne Health, Parkville, VIC, Australia; ^6^Department of Aged & Liaison Psychiatry, Alfred Health, Melbourne, VIC, Australia; ^7^Northwestern Mental Health, Melbourne Health, Melbourne, VIC, Australia

**Keywords:** pharmacogenomics, phenoconversion, CYP2D6, CYP2C19, CYP2C9

## Abstract

**Introduction:** Polypharmacy and genetic variants that strongly influence medication response (pharmacogenomics, PGx) are two well-described risk factors for adverse drug reactions. Complexities arise in interpreting PGx results in the presence of co-administered medications that can cause cytochrome P450 enzyme phenoconversion.

**Aim:** To quantify phenoconversion in a cohort of acute aged persons mental health patients and evaluate its impact on the reporting of medications with actionable PGx guideline recommendations (APRs).

**Methods:** Acute aged persons mental health patients (*N* = 137) with PGx and medication data at admission and discharge were selected to describe phenoconversion frequencies for CYP2D6, CYP2C19 and CYP2C9 enzymes. The expected impact of phenoconversion was then assessed on the reporting of medications with APRs.

**Results:** Post-phenoconversion, the predicted frequency at admission and discharge increased for CYP2D6 intermediate metabolisers (IMs) by 11.7 and 16.1%, respectively. Similarly, for CYP2C19 IMs, the predicted frequency at admission and discharge increased by 13.1 and 11.7%, respectively. Nineteen medications with APRs were prescribed 120 times at admission, of which 50 (42%) had APRs pre-phenoconversion, increasing to 60 prescriptions (50%) post-phenoconversion. At discharge, 18 medications with APRs were prescribed 122 times, of which 48 (39%) had APRs pre-phenoconversion, increasing to 57 prescriptions (47%) post-phenoconversion.

**Discussion:** Aged persons mental health patients are commonly prescribed medications with APRs, but interpretation of these recommendations must consider the effects of phenoconversion. Adopting a collaborative care model between prescribers and clinical pharmacists should be considered to address phenoconversion and ensure the potential benefits of PGx are maximised.

## Introduction

Prescribed medications offer many therapeutic benefits but can also cause serious adverse drug reactions (ADRs). Polypharmacy, often defined as five or more regular medicines, resulting in harmful drug-drug interactions (DDIs), and the presence of genetic variants that strongly influence medication response (pharmacogenomics, PGx), are two well-described risk factors for ADRs (1–4). Pharmacogenomic testing is a strategy to address these risk factors, optimise medication selection and doses, and reduce the burden of ADRs (5). Since many psychotropic medicines have well-established cytochrome P450 2D6 (CYP2D6) and CYP2C19 variants that influence pharmacokinetics, PGx is increasingly being utilised in psychiatry to guide prescribing, particularly for antidepressants. Peer-reviewed PGx dosing guidelines for psychotropics and many other medications are available to help improve the clinical implementation^1^.

In the real-world setting, complexities arise when interpreting PGx results for patients taking medications that can alter the genotype-predicted phenotype of drug metabolising enzymes, a process known as phenoconversion (PC). For example, a genotype-predicted normal metaboliser (NM) for CYP2D6 is expected to have a typical analgesic response to codeine (6). However, if the patient is co-prescribed paroxetine, a strong CYP2D6 inhibitor, the patient's CYP2D6 genotype-predicted phenotype will likely be converted to a poor metaboliser (PM) (7), resulting in greatly reduced morphine formation and diminished analgesia (6). It is more logical in this scenario to follow the codeine guideline recommendation for a CYP2D6 PM rather than the genotype-predicted NM.

Whilst PGx testing offers a useful first step in individualising pharmacotherapy, an overlay of PC could be applied to improve the prediction of medication response for current and planned medication therapy. Due to the added complexity of PC in interpretating PGx results, adopting a novel PGx stewardship program, akin to antimicrobial stewardship, should be considered as a viable strategy to address the challenge (8). This would consist of specific expertise in PGx to ensure that the potential benefits of testing are maximised e.g., clinical pharmacists embedded in general practice (9).

The aim of this analysis was to determine the degree of possible medication-induced PC in a cohort of acute aged persons mental health patients who had PGx testing. These highly complex patients with multiple co-morbidities and polypharmacy were considered a suitable cohort to investigate the potential impact of PC on medications with actionable PGx guideline recommendations (APRs).

## Methods

### Participants

This is a retrospective analysis utilising a sub-set of acute care psychiatric inpatients who participated in a PGx study. In brief, 270 eligible patients and residents were enrolled over a 10-month period, 177 from two aged persons acute mental health units and 93 from long stay residential units. Of these, 170 acute care patients and 82 residential patients underwent PGx screening. The PGx profiles were determined from blood samples or buccal cheek swabs which were taken on average within 1 week of admission. All patients were asked to consent for sample/swab collection and PGx testing, however, if they were mentally incompetent, the ethics committee advised that a consent waiver should be used. A further sub-set of 137 patients with PGx data who had full medication lists at admission and discharge were selected for this analysis to describe PC. Pharmacy medication records were used to determine the medications taken by each patient. Ethical approval for the PGx study was provided by the Melbourne Health Human Research Ethics Committee (approval number 2012.230).

### Genotyping

DNA was extracted from EDTA whole blood samples and from buccal swabs using either QIAamp mini column kits or by the Qiasymphony SP automated platform. *CYP2D6, CYP2C19, CYP2C9*, and *VKORC1* polymorphic sites were detected by iPLEX extension reactions using the Agena MassArray. The number of *CYP2D6* gene copies was detected by qPCR using a 7900HT PCR system. Any copy number variants were confirmed by long-range PCR. Alleles identified included: CYP2D6^*^2, ^*^3, ^*^4, ^*^5, ^*^6, ^*^7, ^*^8, ^*^9, ^*^10, ^*^14A, ^*^14B, ^*^17, ^*^20, ^*^39, and ^*^41; CYP2C19^*^2, ^*^3, ^*^17 and CYP2C9^*^2 and ^*^3. This genotyping panel covered 95% of known variant alleles in Caucasian populations for *CYP2D6*, 99% for *CYP2C19* and 96% for *CYP2C9*. Additionally, common variant alleles were also selected to cover African and Asian populations. It is important to note that the ^*^1 allele is not directly genotyped and is assigned as the “wild type” allele for *CYP2D6, CYP2C19* and *CYP2C9* based on the absence of any interrogated variant in the genotyping panel for each gene. *VKORC1* testing identified the common variant (−1639G > A) in the promoter region of the vitamin K epoxide reductase complex subunit 1 gene (*VKORC1*). This variant has a strong association with warfarin dosage requirements (10).

### Genotype-Predicted Phenotype and Phenoconversion (PC) Assessment for CYP2D6, CYP2C19, and CYP2C9 Enzymes

The *CYP2D6, CYP2C19*, and *CYP2C9* allele activity scores were calculated as previously described (11–14). For each participant, the genotype activity score, which is the sum of activity scores for all alleles in the genotype, was used to assign the phenotype (Supplementary Tables 2, 3). The maximum possible CYP2D6 PC-corrected activity score was calculated by multiplying the genotype activity score by 0 for a strong CYP2D6 inhibitor and by 0.5 for a moderate CYP2D6 inhibitor as previously described (15–17). For example, a CYP2D6 genotype of ^*^1/^*^1 attracts an activity score of 2, which translates to a CYP2D6 NM phenotype. If the patient is taking a strong CYP2D6 inhibitor (e.g., paroxetine), then the PC-corrected activity score becomes 0 (the product of 2 × 0 = 0), which translates to a CYP2D6 PM phenotype. This CYP2D6 PC-corrected activity score system was adapted for CYP2C19 and CYP2C9. In the presence of an inducer, the CYP2C19 and CYP2C9 phenotypes were converted to the next higher activity phenotype (e.g., IM to NM) as previously described (18, 19). Medications were classified as moderate or strong CYP inhibitors or strong CYP inducers based on the US Food and Drug Administration (FDA) table of CYP inhibitors and inducers, the “Flockhart Table,” and a criteria-based classification by Polasek and colleagues (20–22).

### Medications With Actionable PGx-Guideline Recommendations

The Clinical Pharmacogenetics Implementation Consortium (CPIC) and the Dutch Pharmacogenetics Working Group (DPWG) PGx guidelines were used to assess whether a patient's prescribed medication had an APR based on the genotype-predicted phenotype and the PC-corrected phenotype. A medication was considered to have an APR if the CPIC or DPWG guidelines recommended a change in dose or suggested an increased risk of adverse effects. For example, a patient taking citalopram was considered to have an APR if they had a CYP2C19 genotype-predicted phenotype or a PC-corrected phenotype of a PM, where CPIC recommends a 50% reduction in the starting dose (23). Medications that autoinhibit their own metabolism were assessed for APRs using the genotype-predicted phenotype only.

### Statistical Testing

Chi-squared statistical testing was used to describe the differences in predicted phenotype frequencies between the study participants against expected frequencies in the Australian population based on data from our previous study of 5,408 Australians (18). A Wilcoxon signed rank test was used to assess the difference in the number of prescribed medications with APRs between admission and discharge. Statistical analyses were performed using GraphPad Prism version 9.0.2 for Windows, GraphPad Software, San Diego, California USA, www.graphpad.com; and IBM Corp. Released 2020. IBM SPSS Statistics for Windows, Version 27.0. Armonk, NY: IBM Corp.

## Results

### Patient Characteristics

The average age of patients who had PGx testing was 78.5 years (range 60 to 97 years), with 61% female and 39% male. The ethnic breakdown of these patients was: 89% Caucasian, 4% Asian, 2% African and 1% Middle Eastern, while 3% did not have ethnicity recorded.

### CYP2D6, CYP2C19, CYP2C9 Test Results

The predicted phenotype frequencies for CYP2D6, CYP2C19 and CYP2C9 are summarised in Table 1, whilst results for VKORC1 are given in Supplementary Table 1. The phenotype frequencies for CYP2D6, CYP2C19, and CYP2C9 in the study population were similar to the frequencies reported previously for these CYP enzymes in the Australian population (18) (*P* >> 0.05).

**Table 1:** Observed CYP2D6, CYP2C19, CYP2C9 phenotype frequencies.

**CYP2D6 Phenotype**	**N (%)**	**Expected frequency Mostafa et al. (18)**	**CYP2C19 Phenotype**	***N* (%)**	**Expected frequency Mostafa et al. (18)**	**CYP2C9 Phenotype**	***N* (%)**	**Expected frequency Mostafa et al. (18)**
UM	6 (4.4%)	2.8%	UM	8 (5.8%)	4.2%	NM	87 (63.5%)	64.8%
NM	77 (56.2%)	53.2%	RM	34 (24.8%)	25.8%	IM	47 (34.3%)	31.0%
IM	48 (35.0%)	37.6%	NM	56 (40.9%)	39.7%	PM	3 (2.2%)	4.2%
PM	6 (4.4%)	5.7%	IM	33 (24.1%)	26.9%			
			PM	6 (4.4%)	3.1%			

*UM, Ultrarapid Metaboliser; RM, Rapid Metaboliser; NM, Normal Metaboliser; IM, Intermediate Metaboliser; PM, Poor Metaboliser. Phenotype classifications are based on CPIC definitions. CYP2D6 (χ2 = 2.04, DF = 3, P = 0.56); CYP2C19 (χ2 = 2.21, DF = 4, P = 0.70); CYP2C9 (χ2 = 1.79, DF = 2, P = 0.41)*.

### Inhibitors and Inducers of CYP Enzymes Observed at Admission and Discharge

There were nine phenoconverting medications recorded at admission and eight at discharge (Table 2). Collectively, esomeprazole, sertraline and duloxetine represented 71% of the phenoconverting medications at admission and 76% of the phenoconverting medications at discharge.

**Table 2:** List of perpetrator medications responsible for CYP2D6, CYP2C19 and CYP2C9 phenoconversion identified at admission and discharge.

**Medication**	**No. of Patients**		**Inhibitor**	**Inducer**	**Affected Enzyme(s)**
	**Admission**	**Discharge**			
Amiodarone	3	3	✓ (M)		CYP2C9
Carbamazepine	3	3		✓	CYP2C9, CYP2C19
Duloxetine	11	13	✓ (M)		CYP2D6
Esomeprazole	19	17	✓ (M)		CYP2C19
Fluoxetine	2	1	✓ (S)		CYP2D6
			✓ (M)		CYP2C19
Omeprazole	4	4	✓ (M)		CYP2C19
Paroxetine	2	2	✓ (S)		CYP2D6
Sertraline	15	17	✓ (M)		CYP2D6
Terbinafine	1	0	✓ (M)		CYP2D6

*The CYP inhibitor or inducer effect of each medication is listed along with the affected enzyme(s)*.

*(S), Strong inhibitor; (M), Moderate inhibitor*.

### Predicted Phenoconversion Frequencies for CYP2D6, CYP2C19, and CYP2C9 Enzymes

**Figure 1:**
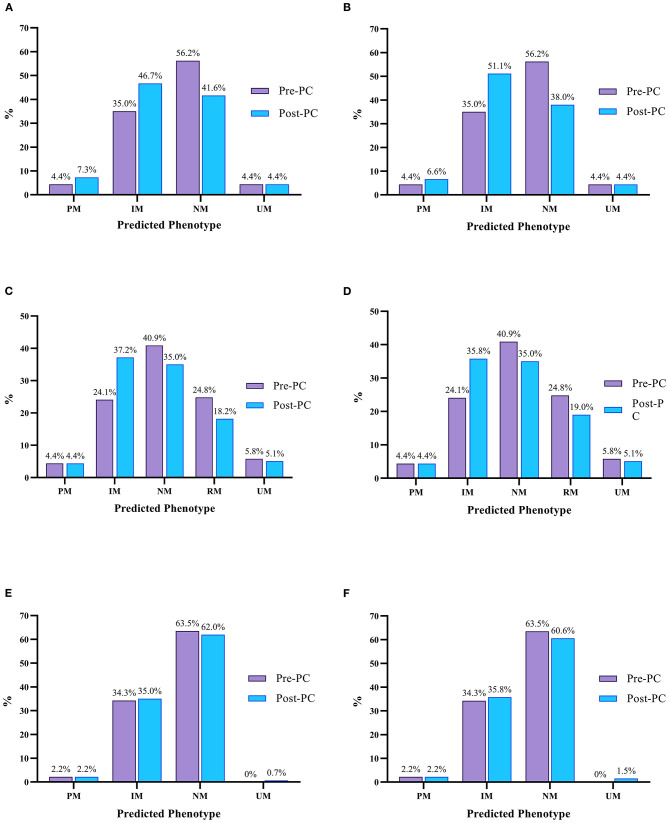
Predicted pre (purple) and post-PC (blue) phenotype frequencies for CYP2D6, CYP2C19 and CYP2C9 at admission and discharge. **(A)** Predicted CYP2D6 Phenotype Frequencies At Admission, **(B)** Predicted CYP2D6 Phenotype Frequencies At Discharge; **(C)** Predicted CYP2C19 Phenotype Frequencies At Admission, **(D)** Predicted CYP2C19 Phenotype Frequencies At Discharge; **(E)** Predicted CYP2C9 Phenotype Frequencies At Admission, **(F)** Predicted CYP2C9 Phenotype Frequencies At Discharge. UM, Ultrarapid Metaboliser; RM, Rapid Metaboliser; NM, Normal Metaboliser; IM, Intermediate Metaboliser; PM, Poor Metaboliser.

Figure 1 shows the predicted phenotype frequencies pre- and post-PC for CYP2D6, CYP2C19 and CYP2C9 at admission and at discharge. CYP2D6 IMs increased by 11.7% at admission and 16.1% at discharge, whilst CYP2C19 IMs increased by 13.1 and 11.7% at admission and discharge, respectively. Only modest changes were noted for other CYP2D6 and CYP2C19 phenotypes, with CYP2D6 PMs increasing by 2.9% at admission and 2.2% at discharge. Due to a small number of CYP2C9 inhibitors and inducers, the CYP2C9 phenoconversion corrected phenotype frequencies were very similar to the genotype predicted phenotype frequencies.

### Medications With APRs

The median number of regular prescription medications recorded on admission was seven (range 1–15) compared to a median of 6 (range 1–13) at discharge. At admission, 103 (75%) patients were categorised as taking polypharmacy (prescribed five or more medications) vs. 89 (65%) patients at discharge. Patients were prescribed 144 different medications at admission, with 19 (13%) having APRs (Table 3). These 19 medications were prescribed a total of 120 times at admission, of which, 50 (42%) had APRs based on genotype predicted phenotype results, increasing to 61 (51%) when PC-corrected phenotypes were considered. At discharge, 143 medications were prescribed, with 18 (13%) having APRs. These 18 medications were prescribed a total of 122 times at discharge, of which, 48 (39%) had APRs based on genotype predicted phenotype results, increasing to 57 (47%) when PC-corrected phenotypes were used (Table 3). A Wilcoxon signed rank test revealed no statistically significant change in prescribed medications with APRs between admission and discharge, *N* = 137, *Z* = 0.258, *P* = 0.796.

**Table 3:** List of medications at admission (A) and discharge (B) with APRs.

**A**.	**Admission**					
**Class**	**Medication**	**Guideline**	**APRs (Pre-PC)**		**APRs (Post-PC)**	
			**Yes**	**No**	**Yes**	**No**
Analgesics	Celecoxib	CPIC	1	2	1	2
	Codeine	CPIC	1	2	1	2
	Meloxicam	CPIC		1		1
Cardiovascular agents	Clopidogrel	CPIC	2	3	3	2
	Metoprolol	DPWG	6	6	10	2
	Warfarin	CPIC	5	8	8	5
Gastrointestinal agents	Omeprazole	CPIC	4		4	
	Pantoprazole	CPIC	16		16	
Psychotropics	Aripiprazole	DPWG		2		2
	Citalopram	CPIC	1	1	1	
	Clomipramine	CPIC		1		1
	Escitalopram	CPIC		4		4
	Haloperidol	DPWG		3		3
	Imipramine	CPIC	1		1	
	Paroxetine	CPIC		2		2
	Risperidone	DPWG	1	18	2	17
	Sertraline	CPIC	2	13	3	12
	Venlafaxine	DPWG	9	4	10	3
	Zuclopenthixol	DPWG	1		1	
	**Total (** ***N*** **=** **120)**		**50 (42%)**	**70 (58%)**	**61 (51%)**	**59 (49%)**
**B**.	**Discharge**					
**Category**	**Medication**	**Guideline**	**APRs (Pre-PC)**		**APRs (Post-PC)**	
			**Yes**	**No**	**Yes**	**No**
Analgesics	Celecoxib	CPIC		2		2
	Codeine	CPIC		1		1
	Ibuprofen	CPIC		1		1
	Meloxicam	CPIC		2		2
Cardiovascular agents	Clopidogrel	CPIC	2	3	3	2
	Metoprolol	DPWG	6	5	10	1
	Warfarin	CPIC	6	8	10	4
Gastrointestinal agents	Omeprazole	CPIC	4		4	
	Pantoprazole	CPIC	15		15	
Psychotropics	Aripiprazole	DPWG		4		4
	Clomipramine	CPIC		1		1
	Escitalopram	CPIC	1	3		4
	Haloperidol	DPWG		1		1
	Paroxetine	CPIC		2		2
	Risperidone	DPWG	2	20	2	20
	Sertraline	CPIC	3	15	4	14
	Venlafaxine	DPWG	8	6	8	6
	Zuclopenthixol	DPWG	1		1	
	**Total (** ***N*** **=** **122)**		**48 (39%)**	**74 (61%)**	**57 (47%)**	**65 (53%)**

*Medication counts are provided pre-PC and post-PC based on the presence (Yes) or absence (No) of APRs. Pre-PC: using baseline genotype results only. Post-PC: using baseline genotype results in addition to phenoconverting effects of concomitant inhibitor/inducer medications*.

## Discussion

Our findings from the analysis of a cohort of acute aged persons mental health patients demonstrated that, (i) the total number of prescribed medications with APRs increased by about 9% at admission and discharge post-PC, (ii) the CYP2D6 PMs and CYP2C19 IMs had the largest increases in PC-corrected phenotypes at admission and on discharge, and (iii) the CYP2D6, CYP2C19, and CYP2C9 phenotype frequencies were similar to the general Australian population. Our approach has recently been highlighted as a practical method to account for the effects of PC on PGx test results (24, 25).

Seventy five percent of patients were taking polypharmacy at admission (defined as five or more regular medications). This is consistent with recent Australian data showing that polypharmacy in Australians >65 years old is as high as 91% and rises with advancing age (26). The prevalence of polypharmacy is also high in older psychiatric patients due to the combination of medication regimens to treat age related co-morbidities plus their psychiatric conditions, thus placing them at greater risk of ADRs and DDIs (27, 28). Therefore, this patient group are likely to benefit significantly from PGx testing and interpretation to optimise their medications.

Using PGx results alone is known to underestimate the incidence of clinically relevant CYP phenotypes (PMs, IMs, UMs). Indeed, one study reported a 5-fold increase in CYP2D6 and CYP2C19 PM frequencies once PC was taken into account (18), while another found a 7-fold increase in CYP2D6 PMs, which was validated by pharmacokinetic sampling (15). While we did not find this degree of increase in PM frequencies, we did observe a notable increase in CYP2D6 PMs−1.7-fold increase at admission and 1.5-fold at discharge. The frequency of CYP2D6 and CYP2C19 IMs was also notably increased—CYP2D6 IMs increased by 1.3-fold at admission and 1.5-fold at discharge, whilst CYP2C19 IMs increased by 1.5-fold at admission and discharge. These results were comparable to a recent study of clozapine treated schizophrenia patients in Australia, which reported a 1.8-fold increase in CYP2D6 PMs and IMs and 1.7-fold increase in CYP2C19 IMs post-PC (25). The lower rates of PC in our analysis compared to other studies may be due to subtle differences in study populations. Here, there were geriatric patients with a mixture of psychiatric disorders on admission with similar ethnic backgrounds, whilst previous studies with higher rates of PC included patients predominantly being treated for depressive disorders receiving fluoxetine, fluvoxamine and paroxetine, well-known strong PC medications (29). This highlights the likely extent and variability of PC and the need to review PC every time there is prescribing or deprescribing of clinically relevant CYP inhibitors and/or inducers.

Regardless, these findings are clinically important as there are many medications which have APRs based on an IM or PM phenotype for CYP2D6 and CYP2C19. In fact, the following medications listed at admission for our study cohort are examples of medicines with APRs for CYP2D6 and/or CYP2C19 PMs and IMs: codeine, clopidogrel, metoprolol, omeprazole, pantoprazole, clomipramine, paroxetine, venlafaxine and zuclopenthixol. Although there was only a modest (8%) increase in prescribed medications with APRs, it still provides useful information that can help optimise dosing and improve the care of this highly complex and vulnerable group of patients. In addition to medication-induced PC, other extrinsic factors have been reported to result in PC including age, frailty, obesity, cancer, inflammation, and vitamin D exposure, all of which are applicable to our cohort of older patients (24). However, presently there are no expert consensus guidelines nor a reliable strategy to quantify and test for the effect of these factors clinically. Thus, further investigation on their impact on PC is warranted.

Our analysis utilised real PGx data and pharmacist reconciled medication profiles, thus providing an accurate reflection of clinical practice. At present, the majority of PGx reports are provided to the treating doctor in a static report format without accounting for the potential effect of PC. This makes it challenging for the prescriber to address PC, especially when the patient's prescribed medications frequently change. For example, if PC is taken into account by the prescriber and the PC-corrected phenotype is used to select the appropriate APRs, then once the patient's “phenoconverting” medication(s) are ceased or changed, the inhibited or induced CYP enzyme will likely revert to baseline function as predicted by the patient's genotype, thus making the APRs corresponding to the PC-corrected phenotype, inaccurate. Additional considerations such as dose and the washout period, determined by the half-life of the phenoconverting drug and the substrate drug, need to be taken into account. This will ensure that dosing of new substrate medications metabolised by the “phenoconverted” CYP enzyme are safely introduced.

Therefore, we propose that a multidisciplinary team with expertise in PGx and clinical pharmacology is required to review PGx test results and the potential impact of PC when making prescribing decisions. In hospitals, a PGx Stewardship (PGS) program could be a feasible and pragmatic approach. The PGS team would advise hospital prescribers on appropriate pharmacotherapy based on PGx test results and PC status. Furthermore, this service could be replicated in the community by a collaborative effort between the General Practice Pharmacist and the GP (9). The community PGS team could address the complex needs of at-risk polypharmacy patients following their discharge from hospital back into primary care (30). Indeed, the biggest impact of the PGS could be in residential aged care facilities, which have recently been highlighted as a major location for polypharmacy, inappropriate prescribing, and lack of dose optimisation resulting in significant patient harm (31, 32).

Current approaches being trialled to address PC include the use of decision support systems to alert the prescriber at the point of prescribing. A recent publication by Bousman et al. describes a new free web-based tool that allows a prescriber or pharmacist to input genotype data and current medications to receive guidance on PC and appropriate PGx-based guideline recommendations (19). In the future, this type of approach may be utilised to address PC by the PGS team at the point of prescribing and dispensing to maximise the benefit of PGx in clinical practice.

This analysis aimed to assess the effect of PC on reporting of medicines with APRs, however, there were several limitations. The PGx testing panel utilised in the study did not include other key pharmacogenes with published CPIC or DPWG PGx-based guidelines (e.g., SLCO1B1, UGT1A1 and TPMT) and therefore the number of medicines with APRs is likely to be under reported. The PGx testing panel covered common alleles in the CYP2D6, CYP2C19, and CYP2C9 genes found in Caucasians, Asians, and Africans, however, some patients may harbour novel variants that will not be detected resulting in incorrect reporting of genotypes. The sample used for this analysis was limited by patients with listed medications at admission and discharge and perhaps a larger cohort is required to better assess the extent of PC on medications with APRs. The PGx study did not adequately assess and collect clinical outcome measures post PGx testing and as such these could not be included in this analysis. The study did not conduct pharmacokinetic (PK) analysis to measure PC due to ethical and clinical challenges with mental health patients. Pragmatically we used currently available best practice references to estimate the maximum possible PC effect based on the concomitant use of CYP inhibitors or inducers. Importantly, this approach does not take into account between patient pharmacodynamic differences that may affect or alter clinical outcome. Therefore, when interpreting the impact of PC clinically, utilising a PGS approach in combination with an understanding of patient response (efficacy and/or toxicity) will be optimal. Future research should be undertaken to understand how the dose and frequency of phenoconverting drugs affect the time course of CYP activity change either by detailed clinical PK studies or physiologically-based PK (PBPK) modelling and simulation (33–36). The latter approach with PBPK is particularly useful to answer questions about genotype-drug-drug interactions in vulnerable groups where PK studies are clinically and ethically challenging.

In conclusion, PGx is increasingly used to inform prescribing in clinical practice. However, PC is a dynamic problem that changes with concomitant medications and should be addressed, especially in older patients taking polypharmacy. We have shown in this analysis that PC can increase the number of APRs that can be applied in clinical practice. Further work is warranted to assess the clinical implications of PC and to establish a suitable approach to resolve this issue in the real-world setting.

## Data Availability Statement

The raw data supporting the conclusions of this article will be made available by the authors, without undue reservation.

## Ethics Statement

The studies involving human participants were reviewed and approved by Melbourne Health Human Research Ethics Committee, Royal Melbourne Hospital, Parkville, Victoria, Australia, 3050. The patients/participants provided their written informed consent to participate in this study.

## Author Contributions

SM wrote the first draft of this manuscript and was responsible for the analysis of the PGx study results. TP and CK contributed to the analysis of results and editing of the article. LS and DH contributed to the design of the original PGx study. All authors revised and approved the final manuscript.

## Conflict of Interest

SM and LS are employees and shareholders of myDNA Inc, a pharmacogenomic testing and interpretation company. TP provides a consultancy service to Sonic Genetics for the interpretation of pharmacogenomic test results. The remaining authors declare that the research was conducted in the absence of any commercial or financial relationships that could be construed as a potential conflict of interest.

## Publisher's Note

All claims expressed in this article are solely those of the authors and do not necessarily represent those of their affiliated organizations, or those of the publisher, the editors and the reviewers. Any product that may be evaluated in this article, or claim that may be made by its manufacturer, is not guaranteed or endorsed by the publisher.

## Footnotes

^1^ https://www.pharmgkb.org/guidelineAnnotations
