# Supplementary material for: Quantifying the Impact of Phenoconversion on Medications With Actionable Pharmacogenomic Guideline Recommendations in an Acute Aged Persons Mental Health Setting
Source: Front Psychiatry. 2021 Aug 19;12:724170. doi: 10.3389/fpsyt.2021.724170 (PMC8416898; doi:10.3389/fpsyt.2021.724170)
Supplement: Supplementary file 1 [file Table_1.DOCX]

**APPENDICES**

The allele and genotype frequencies for VKORC1 in the study cohort are provided in supplementary table 1. The allele frequency for VKORC1 was consistent with expected frequencies in a Caucasian population.

**Supplementary table 1:** Observed allele and genotype frequencies for VKORC1.

| VKORC1 Allele | Predicted Function | N (%) | VKORC1 Genotype | N (%) |
| --- | --- | --- | --- | --- |
| G | Normal warfarin sensitivity | 165(60.2%) | *GG* | 20(14.6%) |
| A | Increased warfarin sensitivity | 109(39.8%) | *AG* | 69(50.4%) |
|  |  |  | *AA* | 48(35.0%) |

**Supplementary table 2** CYP2D6, CYP2C19 and CYP2C9 alleles and activity score.

| CYP2D6 | | CYP2C19 | | CYP2C9 | |
| --- | --- | --- | --- | --- | --- |
| Allele | **Activity score** | **Allele** | **Activity score** | **Allele** | **Activity score** |
| **1XN, *2XN* | 2.0 | **17* | 1.5 | **1* | 1.0 |
| **1, *2* | 1.0 | **1* | 1.0 | **2* | 0.5 |
| **9, *41, *14B, *17, *10XN^#^* | 0.5 | **2, *3* | 0 | **3* | 0 |
| **10* | 0.25 |  |  |  |  |
| **3, *4, *5, *6, *4XN, *3XN* | 0 |  |  |  |  |

#*XN* refers to the number of copies of the CYP2D6 allele (usually two copies), however, there may be more

than two copies. In some cases, technical limitations of the test did not allow measurement of the exact gene copy number.

**Supplementary table 3** CYP2D6, CYP2C19 and CYP2C9 genotypes and predicted phenotypes.

| CYP2D6 | | | | | | | | |
| --- | --- | --- | --- | --- | --- | --- | --- | --- |
| Allele 1 | **Allele 2** | | | **Genotype** | | | **Activity Score** | **Predicted Phenotype** |
| **1* | **1* | | | **1/*1* | | | 2.0 | Normal Metaboliser |
| **1* | **2* | | | **1/*2* | | | 2.0 | Normal Metaboliser |
| **2* | **41* | | | **2/*41* | | | 1.5 | Normal Metaboliser |
| **4* | **41* | | | **4/*41* | | | 0.5 | Intermediate Metaboliser |
| **1XN* | **41* | | | **1XN/*41* | | | 2.5 | Ultrarapid Metaboliser |
| **1* | **10* | | | **1/*10* | | | 1.25 | Normal Metaboliser |
| **2* | **4* | | | **2/*4* | | | 1.0 | Intermediate Metaboliser |
| **3* | **4* | | | **3/*4* | | | 0 | Poor Metaboliser |
| **2* | **10* | | | **2/*10* | | | 1.25 | Normal Metaboliser |
| **1* | **4* | | | **1/*4* | | | 1.0 | Intermediate Metaboliser |
| **2* | **2XN* | | | **2/*2XN* | | | 3.0 | Ultrarapid Metaboliser |
| **1* | **41* | | | **1/*41* | | | 1.5 | Normal Metaboliser |
| **9* | **41* | | | **9/*41* | | | 1.0 | Intermediate Metaboliser |
| **1* | **6* | | | **1/*6* | | | 1.0 | Intermediate Metaboliser |
| **1* | **9* | | | **1/*9* | | | 1.5 | Normal Metaboliser |
| **2* | **2* | | | **2/*2* | | | 2.0 | Normal Metaboliser |
| **1* | **17* | | | **1/*17* | | | 1.5 | Normal Metaboliser |
| **3* | **41* | | | **3/*41* | | | 0.5 | Intermediate Metaboliser |
| **10* | **41* | | | **10/*41* | | | 0.75 | Intermediate Metaboliser |
| **2* | **5* | | | **2/*5* | | | 1.0 | Intermediate Metaboliser |
| **2* | **6* | | | **2/*6* | | | 1.0 | Intermediate Metaboliser |
| **9* | **10* | | | **9/*10* | | | 0.75 | Intermediate Metaboliser |
| **4* | **4* | | | **4/*4* | | | 0 | Poor Metaboliser |
| **2* | **9* | | | **2/*9* | | | 1.5 | Normal Metaboliser |
| **4* | **9* | | | **4/*9* | | | 0.5 | Intermediate Metaboliser |
| **1XN* | **4* | | | **1XN/*4* | | | 2.0 | Normal Metaboliser |
| **2* | **3XN* | | | **2/*3XN* | | | 1.0 | Intermediate Metaboliser |
| **2XN* | **41* | | | **2XN/*41* | | | 2.5 | Ultrarapid Metaboliser |
| **2* | **4XN* | | | **2/*4XN* | | | 1.0 | Intermediate Metaboliser |
| **10* | **14B* | | | **10/*14B* | | | 0.75 | Intermediate Metaboliser |
| **1XN* | **2* | | | **1XN/*2* | | | 3.0 | Ultrarapid Metaboliser |
| **1* | **3* | | | **1/*3* | | | 1.0 | Intermediate Metaboliser |
| CYP2C19 | | | | | | | | |
| Allele 1 | | **Allele 2** | | | **Genotype** | **Activity Score** | | **Predicted Phenotype** |
| **2* | | **2* | | | **2/*2* | 0 | | Poor Metaboliser |
| **1* | | **1* | | | **1/*1* | 2.0 | | Normal Metaboliser |
| **1* | | **2* | | | **1/*2* | 1.0 | | Intermediate Metaboliser |
| **1* | | **17* | | | **1/*17* | 2.5 | | Rapid Metaboliser |
| **17* | | **17* | | | **17/*17* | 3.0 | | Ultrarapid Metaboliser |
| **2* | | **17* | | | **2/*17* | 1.5 | | Intermediate Metaboliser |
| **1* | | **3* | | | **1/*3* | 1.0 | | Intermediate Metaboliser |
|  | |  | | |  |  | |  |
| CYP2C9 | | | | | | | | |
| Allele 1 | | | **Allele 2** | | **Genotype** | | **Activity Score** | **Predicted Phenotype** |
| **1* | | | **1* | | **1/*1* | | 2.0 | Normal Metaboliser |
| **1* | | | **3* | | **1/*3* | | 1.0 | Intermediate Metaboliser |
| **1* | | | **2* | | **1/*2* | | 1.5 | Intermediate Metaboliser |
| **2* | | | **3* | | **2/*3* | | 0.5 | Poor Metaboliser |
| **2* | | | **2* | | **2/*2* | | 1.0 | Intermediate Metaboliser |
| **3* | | | **3* | | **3/*3* | | 0 | Poor Metaboliser |
